# Screening for chronic kidney disease over hospital integration

**DOI:** 10.1002/jgf2.375

**Published:** 2020-09-22

**Authors:** Kei Nagai, Takahiro Hosoi, Kentaro Nakajima, Teiko Arai, Yoshiharu Nakamura

**Affiliations:** ^1^ Department of Nephrology Kamisu Saiseikai Hospital Kamisu Japan; ^2^ Faculty of Medicine University of Tsukuba Tsukuba Japan; ^3^ Department of General Medicine Kamisu Saiseikai Hospital Kamisu Japan; ^4^ Department of Cardiology Kamisu Saiseikai Hospital Kamisu Japan; ^5^ Department of Surgery Kamisu Saiseikai Hospital Kamisu Japan

**Keywords:** chronic kidney disease, creatinine measurement, hospital integration

## Abstract

Hospital integration among rural districts to concentrate medical resources is one of the main projects of the Japanese government. In advance of this, we experienced hospital integration and screening for chronic kidney disease as definitive risk for end‐stage kidney disease and cardiovascular mortality. After that, high‐risk patients have been appropriately referred from generalists to nephrologists and/or cardiologists without acute deterioration of renal function or cardiac sudden death.

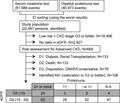

1

To the editor,

With an aging population and a decreasing birthrate, underserved healthcare areas will increase in Japan. Consequently, the concentration of medical resources among such districts is recognized as a major project of the government, which recently designated hospitals for integration.^1^ Our rural district with a population of one hundred thousand has already undergone hospital integration. There are general concerns about worse prognoses and health outcomes due to remote locations,^2^ as hospital integration occasionally forces longer transport times on patients who live in rural areas. Moreover, regarding modest medical services in rural districts, it is indispensable to reduce the social burden of illness by preventing critical organ injury such as ischemic heart disease, stroke, heart failure, and end‐stage kidney disease (ESKD), requiring chronic dialysis and emergency hospitalization.

Chronic kidney disease (CKD) is a definitive and common risk factor for not only development of ESKD and requiring chronic dialysis, but also cardiovascular and all‐cause mortality.^3^ Toward effective identification of populations at high risk for disease burden, we screened patients with CKD diagnosed readily by general physicians with measurable, quantitative, and reliable markers, namely serum creatinine and proteinuria.^4^ This retrospective observational investigation received prior approval by our institutional review board and is publicly registered on UMIN‐CTR. Serum creatinine levels were measured as part of routine clinical practice and health check‐ups, not specifically for this clinical research.

Along with hospital integration in April 2019, we developed a screening system using electronic medical records and establish an algorithm to detect CKD. Among retrospectively obtained data from April 2018 to December 2019, 61 588 creatinine measurements and 40 973 dipstick proteinuria tests were performed in 12 335 men and 9756 women with a mean age of 48 ± 23 (±SD) years. Each participant's worst results for estimated glomerular filtration rate (eGFR) and proteinuria were evaluated. The sequential data from individual patients sometimes contained transient elevations of serum creatinine. CKD is diagnosed based on reduced eGFR persisting for 3 months.^4^ To exclude patients with acute kidney injury (AKI) in this study, the latest data were also assessed to determine whether renal injury had recovered from eGFR less than 30 mL/min/1.73 m^2^ to more than 30 mL/min/1.73 m^2^. If a subject did not have AKI, he or she was regarded as possibly having CKD.

The number of creatinine and urinalysis measurements increased over the period of hospital integration. The mean number of creatinine tests changed from 2839 ± 127 per month before integration to 3234 ± 155 per month after integration and the mean number of urinalyses from 1870 ± 109 to 2184 ± 159 (Figure S1). Among them, 668 patients were found to have an eGFR less than 30 mL/min/1.73 m^2^ that is CKD stage 4 or worse (Figure 1). Of them, 108 had AKI, 133 were on chronic dialysis, 133 died before the end of the investigation, and 76 were transferred to other facilities or were started on conservative terminal care without the option of having chronic dialysis. Moreover, the level of proteinuria is a definitive risk factor for rapid decline in eGFR and development of ESKD.^4^ This screening successfully identified 29 (0.13%) patients with CKD G5 and 62 (0.28%) patients with CKD G4 and with massive proteinuria (A3; 2+ or more) as high risk of disease burden. Since identification, these patients have been appropriately referred from generalists to nephrologists and/or cardiologists without acute deterioration of renal function or sudden cardiac death.

**FIGURE 1:**
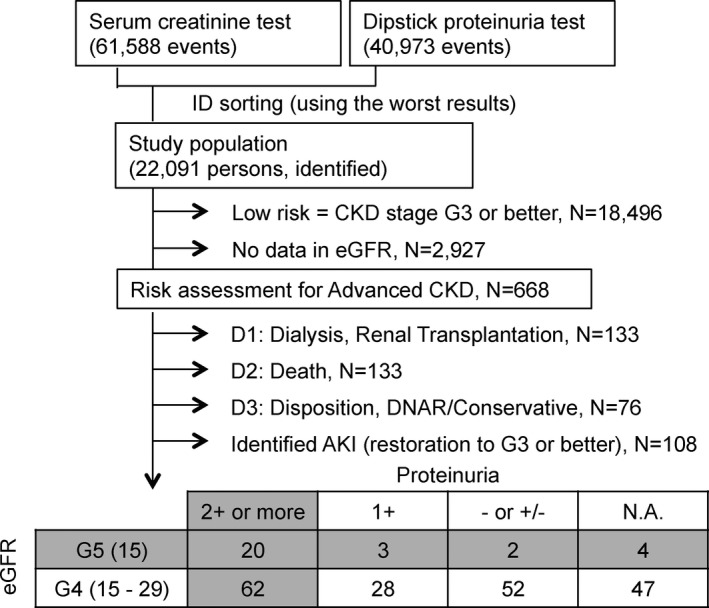
Numbers of chronic kidney disease cases detected by the screening. Obtained results were merged and sorted by identification number (ID). AKI, acute kidney injury; DNAR, do not attempt resuscitation; eGFR, estimated glomerular filtration rate; N.A., not available

As we experienced, electronic medical record screening helps to recognize more high‐risk patients based on CKD diagnosis in an integrated hospital and properly refer them to specialists. Early detection, staging, and appropriate referral for CKD by primary care clinicians are important in reducing the comprehensive burden of disease.^5^ As hospital integration is expected to arise in every region in Japan, diagnosis of CKD may be one of simple tools for early detection and treatment and efficient consultation in the local healthcare setting during medical reorganization.

### CONFLICT OF INTEREST

The authors have stated explicitly that there are no conflicts of interest in connection with this article.

### ETHICAL APPROVAL

This retrospective observational investigation received prior approval by our institutional review board (Kamisu Saiseikai Hospital; #19‐0002) and publicly registered at UMIN‐CTR (#UMIN000039020). Serum creatinine levels were measured as part of routine clinical practice and health check‐ups in Kamisu Saiseikai Hospital, not specifically for this clinical research.

REFERENCES1Website Available from: https://www.mhlw.go.jp/content/10800000/000551037.pdf. Accessed June 1, 2020.2

Bello
AK
, 
Hemmelgarn
B
, 
Lin
M
 et al Impact of remote location on quality care delivery and relationships to adverse health outcomes in patients with diabetes and chronic kidney disease. Nephrol Dial Transplant. 2012;27(10):3849–55.2275938510.1093/ndt/gfs2673

Go
AS
, 
Chertow
GM
, 
Fan
D
, 
McCulloch
CE
, 
Hsu
CY
. Chronic kidney disease and the risks of death, cardiovascular events, and hospitalization. N Engl J Med. 2004;351(13):1296–305.1538565610.1056/NEJMoa0410314

Levey
AS
, 
de Jong
PE
, 
Coresh
J
 et al The definition, classification, and prognosis of chronic kidney disease: a KDIGO Controversies Conference report. Kidney Int. 2011;80(1):17–28.2115087310.1038/ki.2010.4835

Chen
TK
, 
Knicely
DH
, 
Grams
ME
. Chronic kidney disease diagnosis and management: a review. JAMA. 2019;322(13):1294–304.3157364110.1001/jama.2019.14745PMC7015670

## Supporting information

Figure S1

Supplementary Material
