# Supplementary figures and images for: Screening for chronic kidney disease over hospital integration
Source: J Gen Fam Med. 2020 Sep 22;21(6):294–5. doi: 10.1002/jgf2.375 (PMC7689234; doi:10.1002/jgf2.375)

Supporting Figure 1

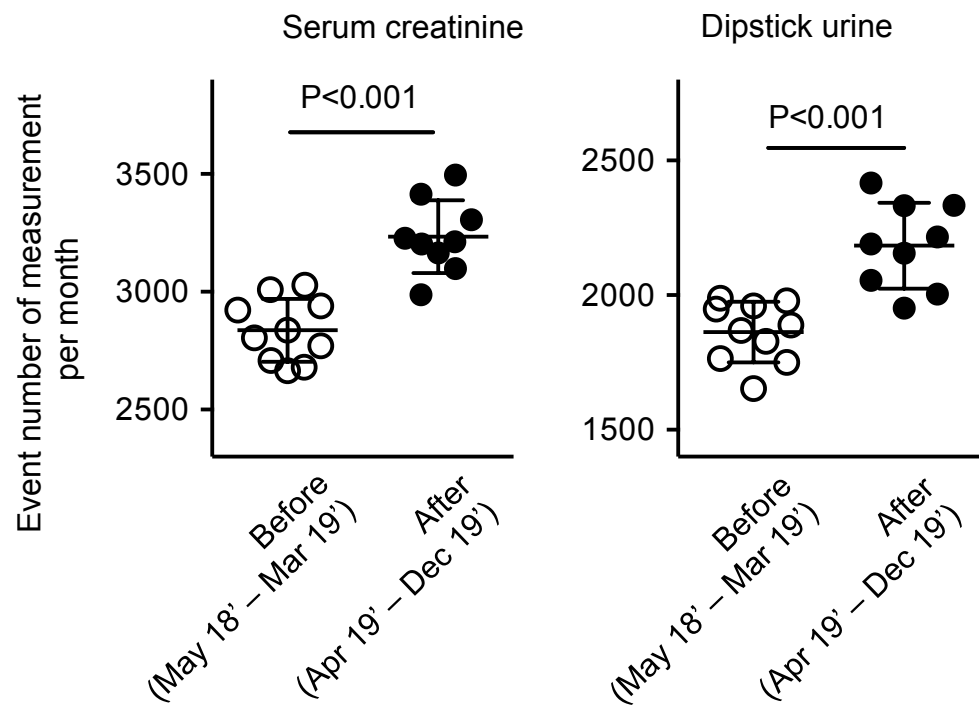

Supplement: Supplementary file 1 — Figure S1 [file JGF2-21-294-s001.pdf]
