# Supplementary material for: Screening for chronic kidney disease over hospital integration
Source: J Gen Fam Med. 2020 Sep 22;21(6):294–5. doi: 10.1002/jgf2.375 (PMC7689234; doi:10.1002/jgf2.375)
Supplement: Supplementary file 2 — Supplementary Material [file JGF2-21-294-s002.docx]

**Figure S1 Serum creatinine and proteinuria measurement over the hospital integration.** Mean numbers of serum creatinine measurements (A) and dipstick proteinuria tests (B) are shown per month before and after hospital integration in April 2019. A circle indicates the number of tests in each month.
